# The new challenge of “exercise + X″ therapy for Duchenne muscular dystrophy—Individualized identification of exercise tolerance and precise implementation of exercise intervention

**DOI:** 10.3389/fphys.2022.947749

**Published:** 2022-08-05

**Authors:** Yuhui Su, Yafeng Song

**Affiliations:** ^1^ Department of Exercise Physiology, Beijing Sport University, Beijing, China; ^2^ Institute of Physical Education, Jilin Normal University, Siping, China; ^3^ China Institute of Sport and Health Science, Beijing Sport University, Beijing, China

**Keywords:** duchenne muscular dystrophy, DMD, exercise, combined therapy, mdx

## Abstract

Duchenne muscular dystrophy (DMD) is an X-linked recessive fatal muscular disease. Gene therapy, cell therapy, and drug therapy are currently the most widely used treatments for DMD. However, many experiments on animals and humans suggested that appropriate exercise could improve the effectiveness of such precision medicine treatment, thereby improving patient’s muscle quality and function. Due to the striated muscle damage of DMD individuals, there are still many debates about whether DMD animals or patients can exercise, how to exercise, when to exercise best, and how to exercise effectively. The purpose of this review is to summarize and investigate the scientific basis and efficacy of exercise as an adjuvant therapy for DMD gene therapy, cell therapy and drug therapy, as well as to present the theoretical framework and optional strategies of “exercise + X″″ combination therapy.

## 1 Duchenne muscular dystrophy overview

DMD is an incurable fatal muscular disease with X-linked recessive inheritance, with an incidence of 1/3,500 male infants. The disease is caused by a mutation in the Dystrophin gene on chromosome Xp21, which results in the deletion or significant reduction of dystrophin protein expression on the sarcolemma. Dystrophin serves as a vital link between the cytoskeleton protein and the extracellular matrix of rhabdomyocytes. Dystrophin deficiency destroys the integrity of the muscle membrane. When muscle cells contract, they lose structural stability and become prone to necrosis. As a result, muscle regeneration ability is depleted and replaced by adipose tissue and connective tissue, resulting in loss of walking ability in boys before the age of 13 and death after adulthood (Zhang and Li, 2018). Protein degradation, apoptosis of the muscle nucleus, persistent muscle cell necrosis, high-shrinkage fiber, and monocyte infiltration were all observed in DMD patients’ muscle pathology. Mdx mice, like humans, lack dystrophin, resulting in muscle degradation and regeneration failure, and can be used as an animal model of DMD to assess the effects of various therapies on muscle malnutrition (Zhang and Li, 2018; Radley-Crabb et al., 2014).

The new treatment strategies used in DMD clinical practice can be divided into three categories: 1) The DMD muscular dystrophy defense mechanism (such as anti-inflammatory treatment, regulating myostatin, and anti-fibrosis). 2) Utrophin protein expression repair, because utrophin can partially replace dystrophin function. 3) Gene therapy (exon jump, senseless mutation, micro/mini-dystrophin gene replacement therapy mediated by adeno-associated virus, CRISPR-Cas9 mediated gene editing, etc.) restores dystrophin protein expression (Li and Liang, 2016). Several existing gene therapies for DMD, such as gene addition, exon jump, stop codon reading, and gene editing, can partially restore functional dystrophin expression. Other cell therapies and drug treatments aim to improve muscle function and quality by addressing the pathogenesis and symptoms of DMD (Liu et al., 2018; Verhaart and Aartsma-Rus, 2019; Zhang et al., 2019). Although DMD is considered incurable, various new treatment methods have promising outcomes, and the best treatment may be a combination of several methods. For example, gene therapy combined with small molecule therapy can be used to restore dystrophin expression and reduce fibrosis. Appropriate sports activities may improve the effectiveness of these precision medical instruments as well as patients’ muscle quality (Kostek, 2019).

One study published in 1966 discovered that when twenty-four patients with muscular dystrophy completed a one-year maximum resistance exercise program, their muscle strength improved significantly in the first 4 months (Vignos and Watkins, 1966). There are still many disagreements among DMD patients about whether or not to exercise, how to exercise, when to exercise, the effect of exercise, and so on (Zhang and Yang, 2012; Shi, 2015; Lei et al., 2020). With the rapid development of DMD therapy and the fact that patients may be active enough at some stages, it is critical to fully comprehend the effect and mechanism of exercise (Kostek and Gorden, 2018). The purpose of this review is to summarize and discuss the scientific basis of exercise as an adjuvant therapy for DMD gene therapy, cell therapy, and drug therapy, as well as to propose a theoretical framework and optional strategies for ‘exercise + X′ combined therapy.

## 2 The theory that exercise is beneficial for Duchenne muscular dystrophy

There have been some significant studies involving exercise in DMD patients. In addition to the aforementioned 1966s study (Vignos and Watkins, 1966), some studies indicated that a low-frequency electrical stimulation(mimicking low-intensity exercise) program of the muscle could substantially increase the torques in the stimulated leg of DMD patients (Zupan, 1992; Zupan et al., 1993). A human trial study discovered that patients with DMD who used whole body vibration exercise (WBVE) (frequency 7–24Hz, amplitude 2–4 mm), bone mass, muscle strength and some bone markers could not be decreased, suggesting that it could be an important choice for DMD rehabilitation (Moreira-Marconi et al., 2017). Maintaining knee strength and joint flexibility would be important factors in the ability to perform walking, climbing and supine to stand activities (Duong et al., 2022). Some rehabilitation guidelines for DMD patients have been published (Grange and Call, 2007; Sejerson and Bushby, 2009; Markert et al., 2011), advocating systematic analysis of muscle function in malnourished animals and patients to determine potential exercise load thresholds to avoid damage, as well as the avoidance of elongated contraction and high resistance training. As a result, lengthening contraction (eccentric contraction) is likely to be a disadvantage for DMD patients, and there is actually a lot of room for developing exercise methods and techniques for DMD patients that are distinct from physical exercise in general.

More research on the benefits of exercise comes from mdx mouse models (Fowler et al., 1990). A 15-weeks endurance swimming program (2 h per day, 5 days per week) was beneficial to the mdx mice, improving muscle regeneration by increasing the proportion of oxidative fibres and decreasing muscle fatiguability, as well as increasing the incidence of soleus fibres with characteristics intermediate to those of fast- and slow-twitch fibre types, implying a possible exercise-induced fibre type transformation as an adaptation to functional demand (Hayes et al., 1993; Lynch et al., 1993; Hayes and Williams, 1997; Hayes and Williams, 1998). A study confirmed that 8 weeks of voluntary running wheel exercise can improve the regeneration ability of exercise mice, the survival rate of Dystrophin positive fibers, and skeletal muscle function by expanding the satellite cell pool without affecting the precursor of muscle pathology (Kogelman et al., 2018). One week of voluntary and voluntary running wheel exercise improved tibialis anterior muscle brittleness in mdx mice, primarily by maintaining muscle cell excitability and activating the calcineurin pathway (Delacroix et al., 2018). Long-term chronic exercise may temporarily offset the damage caused by the work of mdx skeletal muscle by enhancing muscle regeneration and repair. The number of large DRG-groups (degenerative-regenerative groups) in the hind limb muscles of mdx mice was reduced, and the active degenerative regeneration cycle in mdx muscle was shortened (Okano et al., 2005). Low-intensity exercise for 8 weeks reduced oxidative stress levels in brain tissue and gastrocnemius and improved energy metabolism in DMD animals (Hoepers et al., 2020).

The metabolic changes that occurred in the skeletal muscle cells of patients with dystrophy patients and mouse models were both pathogenic (inappropriate body mass changes, mitochondrial dysfunction, reduced adenosine triphosphate (ATP) levels, and increased Ca^2+^) and compensatory (increased phosphorylated AMP activated protein kinase (pAMPK), increased slow fiber numbers, and increased utrophin, among other things) (Heydemann, 2018). 12 weeks of voluntary running wheel exercise increased the expression of utrophin protein, a myodystrophic protein homologue, by 334 ± 63% in quadriceps femoris of mdx mice. Therefore, exercise could be used to intervene in DMD patients to increase utrophin expression (Gordon et al., 2014). Mdx mice’s voluntary movement increased oxidative capacity and autophagy markers even more than healthy mice’s (Hulmi et al., 2013). A 12-week voluntary low-resistance running wheel exercise in mdx mice can lead to skeletal muscle adaptation, as evidenced by improved flexor plantar contractile function, anti-fatigue ability, and mitochondrial adaptation, highlighting the importance of low-intensity exercise as an early therapeutic intervention for children with Duchenne muscular dystrophy (Baltgalvis et al., 2012). Call et al. discovered that after 12 weeks of voluntary running and resistance running training, the grip strength of the two groups of mice increased significantly compared to the normal rearing group of mdx mice, as did the relative tonic contractility of the soleus muscle (Call et al., 2010). Furthermore, Call et al. discovered that 3 weeks of voluntary running improved antioxidant capacity, muscle contractility, total contractile protein concentration, and quadriceps -hydroxyl-coA dehydrogenase activity in 21-day-old mdx mice (Call et al., 2008). Nocetti et al. discovered that 4 weeks of progressive swimming training reduced lipid peroxidation levels in mdx mice’s gastrocnemius, diaphragm, hippocampus, and striatum (Nocetti et al., 2021). These findings show that exercise can improve DMD mice’s muscle strength and endurance, demonstrating the beneficial effect of exercise. Because DMD is an inherited disease caused by a dystrophin gene defect, exercise cannot effectively treat it. However, exercise can help to improve muscle plasticity and oxidative stress ability (Frinchi et al., 2021), preserve function, benefit muscular strength and endurance(Hammer et al., 2022), reduce muscle loss, delay muscle strength decline, or increase homologous protein utrophin expression.

Furthermore, respiratory and circulatory failure are important factors in DMD death, and limb muscle failure is insufficient to cause death. As a result, patients’ respiratory and circulatory systems require special attention. Respiratory training and exercise have been shown in studies on DMD patients and mdx mice to improve respiratory function (Lei et al., 2020). Extensive wheel running improved active tension generation in the mdx diaphragm, the muscle most closely resembling muscles in Duchenne muscular dystrophy patients (Dupont-Versteegden et al., 1994). According to one study, 6 weeks of inspiratory muscle exercise significantly improved inspiratory muscle endurance in DMD patients, and the improvement was more significant with longer exercise duration (Topin et al., 2002). Another 2-year inspiratory muscle exercise program in DMD patients significantly increased maximum inspiratory pressure and 12-s maximum spontaneous ventilation, indicating that long-term inspiratory muscle exercise could improve both inspiratory muscle strength and endurance in DMD patients (Koessler et al., 2001). Voluntary exercise improved hind limb and diaphragm capacity in 4-week-old mdx mice. Respiratory and masticatory muscle training improved functional ability in DMD patients (≥12 years old) (Morici et al., 2017). In mdx mice, moderate and low intensity exercise improved skeletal muscle function, decreased cardiac dysfunction, and improved respiratory ability in a dose-dependent manner. Moderate exercise can reduce the cross-sectional area of fat cells while increasing serum adiponectin levels (Zelikovich et al., 2019). Adiponectin is a hormone that has been shown to have insulin-sensitizing, fat-burning, and anti-inflammatory properties in a variety of tissues, including skeletal muscle. Adiporon, an orally active adiponectin receptor agonist, was shown in one study to have several beneficial effects on dystrophic muscle. This molecule may hold therapeutic promise in the treatment of DMD and other muscle and inflammatory disorders (Abou-Samra et al., 2020).

Furthermore, some exercises can help DMD patients improve their circulatory function. Heart failure claims the lives of 10% to 20% of children with DMD. Yoga practice combined with high-intensity Physical Therapy (PT), particularly family programs, has been shown to improve cardiac function in children with DMD by preserving heart rate variability (HRV) (Pradnya et al., 2019). Voluntary wheel exercise for 8 weeks increased dystrophin expression and thus improved cardiac function in mdx mice without affecting muscle pathology(Kogelman et al., 2018). Call et al. discovered that 3 weeks of voluntary running increased antioxidant capacity, total contractile protein concentration, cardiac citrate synthase, and cardiac -hydroxyl-coA dehydrogenase activity in 21-day-old mdx mice (Call et al., 2008). (The overall situation is shown in Table 1)

**TABLE 1:** A possible mechanism of action between benificial and harmful exercise.

	Benificial exercise	Harmful exercise
muscle regeneration	increase the expression of homologous protein utrophin	telomeres shorten in muscle cells
	promote muscle regeneration, increase muscle strength and bone mass	increase cell apoptosis and the amount of damaged necrotic tissue
oxidative stress	reduce oxidative stress and improve energy metabolism	increase the expression of fast muscle fiber and upregulate oxidative stress
muscle fatiguability	maintain the excitability of muscle cells, activate the calcineurin pathway, and reduce muscle fatiguability	activate Ca^2+^ dependent muscle degradation and increase muscle fatiguability
• inflammatory response	increase the level of serum adiponectin which can sensitize insulin, burn fat, and ameliorate inflammatory disorders	upregulate ECM proteins involved in innate immunity pathways, downstream receptors and signaling molecules, and increase macrophage infiltration

## 3 The theory that exercise is harmful for Duchenne muscular dystrophy

Mdx mice have also been used in studies that suggest exercise is harmful. Lim et al. discovered that mdx mice subjected to 12 h of free running training in a cage had increased apoptosis, as evidenced by a significantly higher proportion of TUNEL staining positive muscle fibers in the soleus muscle and a significantly higher Bax/Bcl-2 ratio (Lim et al., 2004). Another study discovered that telomere shortening was more visible in mdx muscle than in diaphragm, and 12 m/min treadmill exercise caused more telomere shortening than wild-type mice, promoting various pathological events of muscle necrosis (Vita et al., 2020). The sedentary and active mdx mice had 34 different proteins that were responsible for glucose metabolism, energy production regulation, and sarcomere structure maintenance. Exercise caused a typical increase in fast muscle fibrin, accompanied by an increase in several glycolyases, and mdx exercise muscles did not show the metabolic changes associated with the typical fast to slow muscle transition observed in aerobic exercise muscles(Gamberi et al., 2018). Whitchead’s study discovered that after 45 min of downhill running at 10 m/min, the membrane permeability of the extentor digitis longus muscle in mdx mice increased, as evidenced by a significant increase in the proportion of evans blue dye positive muscle fibers. Ca^2+^ entry through tension activated channels and subsequent activation of Ca^2+^ dependent degradation pathways cause increased membrane permeability in tension-induced muscular dystrophy (Whitehead et al., 2006). Coles et al. discovered that 3 weeks of voluntary wheel exercise can aggravate the mdx phenotype by increasing the number of damaged necrotic tissue and macrophage infiltration. Exercise induced additional and larger gene expression changes in mdx mice, according to whole-gene expression profiles, and the most influential pathways were related to immune function or extracellular matrix (ECM) interactions (Coles et al., 2020).

According to current research findings, the harmful effects of exercise may be related to the forced exercise mode, which manifests in muscle cell mechanisms such as increased apoptosis, shortened telomeres, increased expression of fast muscle fiber, increased membrane permeability, Ca^2+^ dependent muscle degradation activation, oxidative stress (Da et al., 2021), enhanced immune response, and so on. These processes may be detrimental to muscle fiber regeneration and quality maintenance in mdx mice. In fact, exercise can cause these muscle fiber responses in normal healthy mice as well as diabetic mice, but it can also improve muscle mass and function (Phaneuf and Leeuwenburgh, 2001). This suggests that these muscle fiber responses are not the key to muscle loss. Due to the low exercise tolerance of mdx mice, controlling the exercise mode and intensity while minimizing harmful exercise may be the key problem of DMD exercise dose control (The overall situation is depicted in Table 1).

## 4 Different theories of exercise for Duchenne muscular dystrophy

There were also findings that activity in mdx mice exerted a beneficial effect on dystrophic skeletal muscle but worsened the heart function(Hourde et al., 2013). Multisystemic investigations, including respiratory and cardiac muscle evaluation, are required to safely prescribe exercise as a therapy to DMD patients (Spaulding and Selsby, 2018). Another study discovered that voluntary exercise may hasten the progression of ventricular dilation and fibrosis in young mdx mice (Costas et al., 2010). Long-term wheel running, on the other hand, harmed diaphragm function while improving cardiac and plantarflexor function in the mdx mouse (Selsby et al., 2013). A closer look at these studies reveals some hints: exercise, whether good or bad, is not closely related to the exercise response of muscle fibers. For example, exercise increases apoptosis and sarcolemma permeability, which has been found in many human and animal studies, but is harmful in mdx mice, and it is not reasonable to attribute this to the exercise response of muscle fibers. These separate studies show that the difference in performance is often due to the exercise itself. Exercise has been proposed as a treatment for DMD, but it has not been universally accepted because it harms malnourished muscles. It can be difficult to draw the precise line between appropriate and inappropriate behavior. The following sections will look at the reasons for the distinction between appropriate and inappropriate practice from a variety of perspectives.

### 4.1 Contraction mode of muscle

Long-term adaptation to eccentric and concentric contractions differs only slightly in a normal healthy population. In DMD patients, however, lengthening contraction caused more muscle cell destruction, cell damage, and inflammation than shortening contraction. As a result, muscle contraction mode may become an important variable in exercise prescription for DMD patients (Kostek and Gorden, 2018). Upper limb training with an arm dynamometer was more effective than ROM training alone in maintaining and improving endurance, arm function, and muscle strength in early DMD patients (Alemdaroglu et al., 2015).

Animal studies have revealed more evidence of differences in muscle contraction. Chronic exercise has little effect on the fitness of wild type muscles, resulting in maladaptation of mdx muscles and an imbalance between protection and injury signals. The loss of dystrophin and the breakdown of the dystrophin-glycoprotein complex affect the mechanical conduction of mdx muscle fibers, causing significant damage. However, when mdx mice were subjected to mild exercise, such as normal cage activity, the muscles displayed a milder phenotype. When the forced chronic treadmill regimen was used, the muscle phenotype became more severe (wild type muscle had no adaptive effect). Mild exercise can increase the expression of inSirt1/PGC-1, which improves the protective slow gene fingerprint (MHC1 and SERCA2). This response can achieve efficient proliferation as well as appropriate inflammation control, eventually leading to a milder phenotype. The injury path remained the same when the forced chronic treadmill program was used. The low mechanical transduction threshold described above causes maladaptation, with a significant decrease in the expression of protective pathways involved in mechanical metabolic coupling (including further autophagy damage, known as Bnip3). This process eventually results in impaired regeneration and depletion of endogenous anti-inflammatory signals, which may contribute to the establishment of chronic and unbalanced inflammation and a more severe phenotype. The ability to adapt to exercise-protective metabolic oxidative pathways, in particular, is impaired, whereas gene expression of proteins involved in injury signaling, such as oxidative stress and inflammation, remains upregulated (Camerino et al., 2014).

(Lou, 2012). A researcher demonstrated that independent running in the cage for 30 min per day for 14 days caused muscle fiber necrosis and sarcolemma injury in mdx mice, as evidenced by a significantly increased proportion of ring-dead regenerated fibers, central nuclear fibers, high-contraction fibers, and ebd-positive muscle fibers of the triceps calf muscle. Short contractions without resistance caused only minor damage to dystrophin-deficient muscles and significantly reduced the occurrence and pathological changes associated with muscle fiber necrosis in mdx mice. As a result, it is recommended that DMD patients avoid elongated and isometric contractions and instead perform more short contractions without resistance to avoid muscle injury and disuse atrophy. The main cause of muscle injury in mdx mice may be muscle membrane damage caused by shearing force during muscle contraction. Different patterns of contraction generate different shear forces, which cause varying degrees of myomembrane injury and decreased muscle contraction ability. (Lou, 2012).

Another study discovered that after 45 min of continuous downhill running and horizontal platform running, the hind leg muscles of mdx mice were significantly damaged, whereas horizontal running significantly activated the muscles of mdx mice, but the damage to the muscles was very light and recovered quickly. It has also been discovered that during regular cage activity and exercise, the medial calf muscle may be more vulnerable to contraction-induced muscle injury. Because these muscles are mostly made up of rodent fast muscle fibers, they are more prone to injury. Further research into the functional role of these muscles in downhill running and their susceptibility to muscle injury in mdx mice is warranted (Mathur et al., 2011).

The above studies show that the more beneficial ways of exercise are voluntary and low-load exercise, such as voluntary cage running, swimming and inspiratory muscle exercise. The more harmful form of exercise is downhill running, which increases the lengthening contraction of exercise.

### 4.2 Exercise duration

The duration of an exercise program includes two components: the length of a continuous exercise and the number of years of program implementation. Internal muscle fibers had enough time to play a role in the compensation mechanism of injury in studies that lasted from 6 weeks to 2 years (Koessler et al., 2001; Call et al., 2008; Call et al., 2010). These studies, however, only used muscle strength and endurance-related indicators to evaluate the effect of exercise, without controlling for compensatory factors (regeneration of muscle fibers, proportion increase of type I muscle fibers, sarcomere consistency and so on). As a result, the findings of these studies are influenced by “compensation” confounding factors, and cannot completely rule out the possibility of muscle injury caused by exercise. The duration of exercise in studies showing that exercise is harmful is short, ranging from 5 min to 3 days (Brussee et al., 1997; Vilquin et al., 1998), and all of them use indicators that can directly reflect muscle fiber damage. TUNEL positive muscle nucleus, Bax/Bcl-2 ratio, sarcolemma injury (EBD positive muscle fiber ratio, serum creatine kinase, -galactosidase), T2-weighted magnetic resonance imaging (ratio of increased pixels of T2 value in muscle magnetic resonance), and other indicators are examples. The comparison demonstrates that whether exercise is beneficial or detrimental is closely related to exercise adaptation. It is consistent with conventional wisdom that the negative effects of exercise are often observed after acute exercise or in the early stages of exercise regimens, whereas the positive effects of exercise are observed during longer periods of exercise regimens. Exercise’s beneficial effects necessitate the accumulation of “muscle damage,” which was also observed in mdx mice.

### 4.3 Exercise intensity

Exercise intensity is a important consideration in exercise prescription, particularly for DMD patients or mdx mice with extremely low exercise tolerance. When mdx mice were exercised at 12 m/min, their phenotype deteriorated significantly (high intensity). Mdx mice exercised with treadmill regiments of 8 m/min (moderate intensity) and 4 m/min (low intensity) showed improvements in skeletal muscle function, reduced cardiac dysfunction, and improved respiration, usually in a dose-dependent manner. Moderate exercise can decrease adipocyte cross-sectional area while increasing serum adiponectin levels without increasing myometral injury or necrosis, implying that adiponectin could be a useful biomarker for DMD exercise (Zelikovich et al., 2019). Kostek’s exercise intervention study on DMD in animals and humans discovered that high-intensity and long-term eccentric muscle contraction aggravated the disease’s pathological manifestations, whereas lower-intensity and volume cycling training, weight-bearing walking, and water sports did not damage the muscles and may be beneficial. More long-term studies, however, are required due to the small sample size (Kostek and Gorden, 2018). Morroni et al. discovered that running mdx mice at a moderate pace for 1 h twice a week for 8 weeks accelerated cardiac pathological progression, as evidenced by early fibrotic deposition, increased necrosis and inflammation, and decreased cardiac function (Morroni et al., 2021). Duchenne muscular dystrophy (DMD) affected only a subset of skeletal muscle fibers that were specialized for fast contraction (Webster et al., 1988). Another study found that low-intensity training was associated with lower oxidative damage as measured by carbonylation and higher expression of proteins involved in energy metabolism and muscle contraction (Hyzewicz et al., 2015a). Running on a motorized Rota-Rod for 5 days per week for 6 weeks significantly reduced inflammatory-necrotic areas in both the gastrocnemius and quadriceps muscles (Frinchi et al., 2014). Despite the fact that these few studies emphasize the importance of exercise intensity (Lott et al., 2021), in fact, there are great operational barriers. The definitions of high and low intensity are completely different in different exercise modes and exercise objects. Patients with DMD who have poor exercise tolerance should be especially cautious. As a result, there is still a lot of room for research in this area.

### 4.4 Experimental subject

DMD gene therapy improved muscle mass and function in mdx mice significantly. However, clinical conversion did not produce the desired results in human patients. This is due in large part to the fact that mdx mice exhibit the smallest and mildest symptoms of muscular dystrophy, and they react to the gene carrier completely differently than human patients. Furthermore, mice are hundreds of times smaller than boys, making gene therapy on mouse models impossible to scale up. These restrictions do not apply to canine DMD models (cDMD). DMD dogs exhibited more muscular dystrophy symptoms than mdx mice, with animals with greater absolute muscle abundance and mass showing more weakness in DMD models. This is especially true for people with DMD. As a result, cDMD dogs are regarded as a valuable model for clinical DMD gene therapy transformation (Duan, 2015). This exemplifies the ambiguous relationship between DMD efficacy and the target.

Exercise therapy discovered the same problem in mouse models, and the efficacy of exercise depended on the mice’s gender, age, and muscle type. Female mdx mice were found to be more likely than males to develop heart problems after voluntary wheel running in a study using mdx mice (Ferry et al., 2015). Another way to assess mice fitness is to measure the properties of specific muscles. However, depending on the muscle type, the results can vary greatly. The hind limb and diaphragm phenotypes of mdx mice improved when training began before 7 weeks of age, but deteriorated when training began later. However, when 4-week-old mdx mice began training, cardiac performance deteriorated (Hyzewicz et al., 2015b). DMD worsens with age, and muscle mass and function vary greatly. The active participation of muscles is what distinguishes exercise therapy. According to studies, there was no statistically significant difference in the correlation between whole body motor function and whole body muscle strength and age in preschool (45 years and 11 months) DMD patients, whereas lower limb function and muscle strength decreased significantly with age in school-age (6–12 years old) DMD patients. This suggests that DMD patients’ lower limb function and strength began to deteriorate significantly around the age of six (Chen et al., 2022). As a consequence, DMD patients who exercise earlier may benefit more. When muscles are unable to support active movement, exercise therapy may be unavailable or forced to aggravate muscle damage. Furthermore, DMD is particular to the muscles involved. The skeletal muscles are severely affected, but the extraocular and internal laryngeal muscles are unaffected. Mild endurance exercise improved limb skeletal muscles but not the diaphragm in mdx mouse models. Based on the diaphragm’s lower inflammation and repair mechanism, it is hypothesized that at least mild endurance training could help with this muscle deficiency (Morici et al., 2017). This appears to imply that genetic defects are not a determinant of muscular dystrophy. In the presence of genetic defects, there is a lot of room for saving or maintaining muscle mass and function. When implementing exercise therapy, many details of DMD subjects need to be considered in order to develop an effective exercise program; otherwise, exercise therapy may be counterproductive.

### 4.5 Statistical problem

Statistical factors are also the objective reasons for the difference in exercise. In specific independent studies, both the benefits and harms of exercise are statistically determined, and statistical parameters directly influence the choice of theoretical assumptions. For example, the experimental and control groups’ muscle strength test results were 32.33 ± 3.35 and 30.86 ± 3.35 (mean ± standard deviation) respectively, and the statistical results were completely different when the sample size was changed. According to Gianola et al., the benefits of muscle exercise, which have been reported in most studies, may be due to the small sample size and that “harmful effects are still possible.” As a result, despite decades of research, physicians are still unable to directly answer patients with muscular dystrophy’s exercise questions (Gianola et al., 2013). DMD exercise intervention studies currently lack consistency, and clinical studies with larger samples are the only way to avoid statistical bias and establish a unified view.

## 5 “Exercise + X″ therapy vs other treatments for Duchenne muscular dystrophy

DMD treatments are broadly classified into the five technical categories listed below. A. The muscle tissue of patients is induced to produce dystrophin with partial function using adeno-associated virus (AAV) as the carrier of micro-dystrophin introduction, exon hopping therapy, termination codon reading therapy, and CRISPR technology gene correction (Duan et al., 2021). The issue that must be addressed is how to avoid or reduce gene off-target mutation. B. Increased replacement gene expression to replace dystrophin, with a current emphasis on autosomal homologous utrophin gene introduction (Song et al., 2019). The main challenges that these gene therapies face right now are vector immunogenicity and systemic delivery, so the therapeutic effects are highly uncertain. C. DMD cell therapy. The role of myosatellite cells in disease progression in DMD patients remains unknown. It is widely assumed that repeated muscle degeneration and regeneration exhausts muscle stem cells and satellite cells, resulting in a loss of regenerative capacity. As a result, while the use of patient-derived pluripotent stem cells provides unlimited autologous stem cell potential for DMD treatment, its long-term safety and efficacy remain unclear. D. Address malnutrition’s downstream effects, such as membrane instability, intracellular calcium accumulation, poor vascular perfusion, oxidative stress, protein nitrosation, malnutrition, and fibrosis. Long-term glucocorticoid therapy is the gold standard of care for DMD patients (Yang and Lv, 2017). However, because it is accompanied by a number of long-term hormone use side effects (osteoporosis, weight gain, abnormal behavior, diabetes symptoms), it can only slow the progression of the disease. F. Therapy through exercise. Due to the different types of muscle fibers, exercise intensity, and exercise mode, DMD exercise therapy has different effects of exacerbating muscle injury or improving muscle function. As a result, exercise therapy for DMD patients has been a source of contention, and more precise exercise prescriptions are required for intervention.

Combination therapy is a common clinical strategy for treating complex diseases(Cordova et al., 2018). It is worth considering how to combine the above five DMD therapies and their feasibility. According to the findings in Table 2, gene therapy, cell therapy, and some drug therapy have positive effects in mouse models; however, there is a significant difference in exercise therapy, and the safety and effectiveness of voluntary running wheel exercise may be superior. In mdx mice treated with micro-dystrophin gene therapy and dual AAV gene therapy, the difference between “exercise + X” combined therapy was greater, and exercise actually caused side effects (Zhang et al., 2013; Rodgers et al., 2020). Exercise has a synergistic therapeutic effect with Pentoxifylline(Rolland et al., 2006). Combining exercise with AMPK/PPAR agonists improved animal function, decreased serum CK levels and increased oxidative metabolist-related protein expression in mdx mice. Therefore, exercise can be used in conjunction with other treatments for muscular dystrophy(Bueno et al., 2012). While Table 2 provides the scientific basis and feasibility of “exercise + X”therapy, there is one important point to note: the majority of these data are from mdx mice, with a few from DMD dogs and patients. Due to the objective existence of exercise differences (as previously stated), the system construction of “exercise + X″ therapy should be approached from three perspectives: efficacy, method and mechanism. The first is whether the efficacy is significant for DMD, the second is whether the method is feasible, and the third is the mechanism. The question of curative effect has already been raised, and the method’s feasibility and mechanism are primarily discussed here.

**TABLE 2:** Efficacy comparison of major DMD therapies.

Therapy	Object	Method	Result	References
Dystrophin gene therapy	DMD boy	The AAV2.5 vector was injected into one arm’s biceps brachii muscle	All patients had recombinant AAV genomes but no cellular immune response. AAV2. 5, the carrier is non-hazardous and well tolerated	Bowles et al. (2012)
micro-dystrophin gene therapy	GRMD dog	The rAAV2/8-micro-dystrophin expression vector was injected intravenously	The symptoms of muscular dystrophy were relieved, and there was no toxicity or adverse immune effects from rAAV administration	Le Guiner et al. (2017)
micro-dystrophin	mdx/mTR (G2) mouse	Systemic injection of rAAV6-micro- dystrophin	Mice’s muscle force was increased	Ho et al. (2018)
gene therapy				
Utrophin gene therapy	mdx	AAV-Utro intravenous injection	Histological and physiological lesions of muscular dystrophy could be avoided in mouse and large dog models	Song et al. (2019)
	mouse			
	GRMD dog			
Autologous cell therapy	mdx/SCID mouse	Dystrophin was transfected with a monoclonal antibody and implanted into a mouse’s muscle or artery	The elastic properties of 80% muscle fibers were restored to wild-type performance, which was more fatigue resistant	Iyer et al. (2018)
Exosome mediated cell therapy	mdx	Cardiac injection of cardiac progenitor cells (CDCs)	CDCs and their exosomes temporarily restored the expression of partial full-length dystrophin in mdx mice	Aminzadeh et al. (2018)
	mouse			
Prednisone	DMD boy	Prednisone 0.75 mg/kg per day was administered on a 10 days on/10 days off basis	When compared to historical controls, prednisone 10 on/10 off has fewer side effects and extends the ambulant phase by 1 year	Straathof et al. (2009)
Pharmaco therapy				
Prednisone Pharmaco therapy	DMD boy	two prednisone schedules (daily 0.75 mg/kg/day and weekend 10 mg/kg/wk), 12 months	Over a 12-month period, weekend prednisone dosing was as safe and effective as daily prednisone in preserving muscle strength and preventing body mass index increases in boys with DMD.	Escolar et al. (2011)
Idebenone	mdx	4 weeks to 10 months old, 200 mg/kg body weight	It improved voluntary running ability, prevented heart failure, reduced cardiac inflammation and fibrosis, and improved cardiac diastolic dysfunction	Buyse et al. (2009)
Pharmaco therapy	mouse			
Laminin-111pharmaco therapy	GRMD dog	The protein Mslam-111 was injected into the craniotibial muscle	It increased muscle fiber regeneration and repair, enhanced muscle strength and reduced muscle fibrosis	Barraza-Flores et al. (2019)
sActRIIB-Fcpharmaco therapy	mdx	Soluble activin receptor sactriib FC was injected to block myostatin	Muscle mass increased	Hulmi et al. (2013)
	mouse			
Resveratrol traditional Chinese medicine therapy	mdx	4 weeks of age,5 mg/kg bw/day and 15 weeks of treatment	The expression of immune cell markers CD86 and CD163 decreased, and muscle necrosis was alleviated.	Woodman et al. (2021)
	mouse			
Voluntary wheel running	mdx	8 weeks of voluntary wheel running (3–4 days a week)	Prolonged skeletal muscle suspension time and increased dystrophin expression had a positive effect on skeletal muscle and myocardial function	Kogelman et al. (2018)
	mouse			
Voluntary wheel running	mdx	12 weeks of voluntary low resistance wheels	Central nucleus muscle fiber decreased, skeletal muscle contraction function improved and fatigue decreased, and there was no sign that exercise was harmful	Baltgalvis et al. (2012)
	mouse			
Voluntary wheel running	mdx	7 weeks of voluntary wheel running	The oxidative capacity and autophagy markers of skeletal muscle was increased, and were even higher than in healthy mice	Hulmi et al. (2013)
	mouse			
Treadmill training	mdx	Running at a speed of 12 m/min for 30 min, twice a week, with a 48-h or 72-h interval (12 times in total)	Impaired muscle regeneration and the failure of endogenous anti-inflammatory signals exacerbate chronic inflammation and result in more severe muscle phenotypes	Camerino et al. (2014)
	mouse			
Treadmill training	mdx	For 6 weeks, 5 days per week, use the treadmill to gradually increase the load	There was no significant improvement in diaphragm	Morici et al. (2017)
	mouse			
Treadmill training	mdx	4 m/min or 8 m/min, three times a week, 30 min each time, for a total of 6 months	Improved muscle rigidity and strength, respiratory capacity and cardiac function; decreased the cross-sectional area of adipocytes	Zelikovich et al. (2019)
	mouse			
Progressive swimming	Progressive swimming	Four times a week for a total of 4 weeks, 15 min per day in the first week, 20 min per day in the second week, and 30 min per day in the third and fourth weeks	The levels of lipid peroxidation in gastrocnemius, diaphragm, hippocampus and striatum were significantly decreased	Nocetti et al. (2021)
micro-dystrophin	mdx	rAAV6:µDysH3 and rAAV6:µDys5	Both rAAV have the same dose response to prevent muscle damage caused by repeated high-intensity exercise	Rodgers et al. (2020)
exercise+	mouse	Gene therapy + repeated high-intensity exercise		
gene therapy				
A pair of AAVs exercise+ gene therapy	mdx	A pair of AAV vectors were injected into the caudal vein to express nNOS bound dystrophin gene	It improved histopathology, increased muscle strength and prevented eccentric contraction injury and muscle strength decline caused by chronic exercise	Zhang et al. (2013)
	mouse			
	mdx4cv			
	mouse			
Exercise +	mdx mouse	Running at a speed of 12 m/min for 30 min, twice a week for 4–8 weeks. Pentoxifylline (50 mg/kg/day) was injected during exercise	The Ca^2+^channel activity in mdx mice treated with exercise + pentoxifylline was similar to that in wild-type mice	Rolland et al. (2006)
pentoxifylline				

First, a factor to consider in human DMD patients is single nucleotide gene polymorphism (SNP), which is determined by differences in exercise subjects. SNPS can be used to create more detailed classifications of disease types in order to better predict which types will respond to specific treatments. SNP is likely to interact with therapeutic exercise. Many SNPS have been identified that influence exercise response in both healthy and sick people. As a result, certain boys with DMD or BMD may respond positively to increased physical activity, and it is likely that in the future, boys with specific SNPS will respond better to specific gene therapy and specific exercise interventions when combined with other therapies. These synergistic combinations are a prime example of precision medicine’s future (Kostek, 2019).

Second, the type of muscle contraction is critical. Eccentric contractions can cause microtearing, sarcolemma leakage and repair, as well as acute and chronic effects on muscle satellite cell division. As a result, exercise therapy and cell therapy should be combined properly. Furthermore, the opening and resealing of the sarcomembrane during exercise, in addition to transmitting signals of inflammation, growth, and repair, is likely to affect the absorption of the imported virus into both dividing and non-dividing cells, and have significant long-term effects on the effects of such gene therapy (Kostek, 2019).

Third, exercise has an impact on the efficacy of other therapies. Exercise has been shown to influence gene and protein expression of dystrophin, utrophin, and other structural proteins, as well as many signaling pathways in muscle that are directly involved in gene splicing. Furthermore, exercise can affect drug uptake or the working ability of drugs alone, and the correct dose of exercise is likely to increase drug working ability (Kostek, 2019). Exercise response is perhaps the most difficult to predict of all the therapies mentioned above, and in cell therapy, exercise affects the ability of cells to locate, integrate, and organize correctly during exercise-induced stress. Exercise influences the production of local and systemic growth factors, and it is also necessary for proper nervous system innervation (Kostek, 2019). And, as a foundation for new treatments and drug development, the way exercise interacts with the immune system, on which the success of these therapies is dependent, appears critical.

## 6 Summary

Most studies on DMD exercise intervention tend to suggest that DMD patients should avoid stretching and isometric contractions as much as possible, and instead focus on low-intensity short-shortening contractions without resistance to avoid muscle damage and disuse atrophy. However, little is known about exercise rehabilitation therapy for DMD patients, including which patients can exercise and when they exercise, due to differences in exercise caused by muscle contraction mode, exercise duration, exercise intensity, exercise subject (gene polymorphism) and insufficient sample size. In addition to determining the appropriate exercise intensity, a complex and meticulous monitoring of skeletal and cardiac muscle is required. More studies in the future are expected to link fine exercise programs with molecular monitoring, compare differences between animals and humans, and prescribe targeted combined prescriptions for DMD patients with “exercise + X″ therapy. Due to DMD patients’ unique exercise tolerance, independent muscle exercise physiology studies on DMD are required to better understand the effects of muscle activity on skeletal muscle, respiration, and circulation, in order to minimize muscle damage during exercise and improve DMD patients’ respiratory and circulation function.
